# External targeted navigation of ultra-small iron-oxide (U/SPIO) nanoparticles by an external permanent magnet - proof-of-principle as a prerequisite for magnetic drug delivery using U/SPIO

**DOI:** 10.1186/1532-429X-17-S1-P72

**Published:** 2015-02-03

**Authors:** Michael Bietenbeck, Verena Hoerr, Harald Kugel, Cornelius Faber, Ali Yilmaz

**Affiliations:** 1 Department of Cardiology and Angiology, University Hospital Münster, Münster, Germany; 2 Translational Research Imaging Center, University Hospital Münster, Münster, Germany

## Background

Recently, we could demonstrate in humans that (ultra-small) superparamagnetic iron-oxide (U/SPIO)-based contrast agents enable a detailed characterization of myocardial infarct pathology. Considering the multi-functionality of U/SPIO-based nanoparticles (magnetic targeting as well as diagnostic imaging properties) and their superior safety profile compared to gadolinium-based compounds, we performed ex vivo analyses and evaluated the magnetic navigating/targeting properties of U/SPIO.

## Methods

Using a self-constructed closed circuit with a manually tunable pump and an external permanent magnet, the targeting properties of ferucarbotran (U/SPIO) were evaluated. Using a concentration of ferucarbotran similar to the allowed concentration for human use, our self-constructed system allowed us to modify the following parameters: a) the distance between our circuit and the permanent magnet, b) the velocity rate of circulation in our closed circuit and c) the duration of permanent magnet exposure. Drawing off of fluid from our circuit at the location of the permanent magnet allowed us to measure the concentration/accumulation of ferucarbotran using a 9.4-T magnetic resonance scanner and T2*-weighted sequences.

## Results

Prior to starting the pump of our closed circuit and to introducing the magnet, the measured T2* value of our ferucarbotran-solution was 35.7ms (using an iron concentration of 5µg iron/ml water in our circuit). Using a velocity rate of 20cm/sec, a magnet exposure duration of only 5min and a magnet distance of 0 resulted in a T2* value of 15.3ms - indicating successful accumulation of iron particles. Increasing the distance of the magnet to 10mm (while keeping other variables constant) lead to an increase in T2* to 28.2ms - indicating a reduced accumulation of iron particles. Repeated measurements with modified parameters showed a) a (relative) increase in T2* when the distance of the magnet was increased, b) no relevant change in T2* when the velocity rate of circulation was changed between 5-35cm/sec and c) a (relative) decrease in T2* when the magnet exposure duration was increased.

## Conclusions

External targeted navigation of U/SPIO (approved for human use) by an external permanent magnet is technically possible. U/SPIO may play an important future role in magnetic drug delivery and simultaneous diagnostic imaging and therapy surveillance.

## Funding

None.

